# Magnetic Resonance Elastography Shear Wave Velocity Correlates with Liver Fibrosis and Hepatic Venous Pressure Gradient in Adults with Advanced Liver Disease

**DOI:** 10.1155/2017/2067479

**Published:** 2017-04-05

**Authors:** Ahmed M. Gharib, Ma Ai Thanda Han, Eric G. Meissner, David E. Kleiner, Xiongce Zhao, Mary McLaughlin, Lindsay Matthews, Bisharah Rizvi, Khaled Z. Abd-Elmoniem, Ralph Sinkus, Elliot Levy, Christopher Koh, Robert P. Myers, G. Mani Subramanian, Shyam Kottilil, Theo Heller, Joseph A. Kovacs, Caryn G. Morse

**Affiliations:** ^1^National Institute of Diabetes and Digestive and Kidney Diseases, Biomedical and Metabolic Imaging Branch, Bethesda, MD, USA; ^2^National Institute of Diabetes and Digestive and Kidney Diseases, Liver Diseases Branch, Bethesda, MD, USA; ^3^Laboratory of Immunoregulation, National Institute of Allergy and Infectious Diseases, Bethesda, MD, USA; ^4^Critical Care Medicine Department, NIH Clinical Center, AIDS Section, Bethesda, MD, USA; ^5^Division of Infectious Diseases, Department of Microbiology and Immunology, Medical University of South Carolina, Charleston, SC, USA; ^6^Laboratory of Pathology, National Cancer Institute, Bethesda, MD, USA; ^7^National Institute of Diabetes and Digestive and Kidney Diseases, Office of the Director, Bethesda, MD, USA; ^8^Biomedical Engineering, Imaging Sciences and Biomedical Engineering Division, Kings College, London, UK; ^9^Interventional Radiology, NIH Clinical Center, Bethesda, MD, USA; ^10^Gilead Sciences, Inc., Foster City, CA, USA

## Abstract

*Background.* Portal hypertension, an elevation in the hepatic venous pressure gradient (HVPG), can be used to monitor disease progression and response to therapy in cirrhosis. Since obtaining HVPG measurements is invasive, reliable noninvasive methods of assessing portal hypertension are needed.* Methods.* Noninvasive markers of fibrosis, including magnetic resonance elastography (MRE) shear wave velocity, were correlated with histologic fibrosis and HVPG measurements in hepatitis C (HCV) and/or HIV-infected patients with advanced liver disease enrolled in a clinical trial of treatment with simtuzumab, an anti-LOXL2 antibody.* Results.* This exploratory analysis includes 23 subjects: 9 with HCV monoinfection, 9 with HIV and HCV, and 5 with HIV and nonalcoholic steatohepatitis. Median Ishak fibrosis score was 4 (range 1–6); 11 subjects (48%) had cirrhosis. Median HVPG was 6 mmHg (range 3–16). Liver stiffness measured by MRE correlated with HVPG (*r* = 0.64, *p* = 0.01), histologic fibrosis score (*r* = 0.71, *p* = 0.004), noninvasive fibrosis indices, including APRI (*r* = 0.81, *p* < 0.001), and soluble LOXL2 (*r* = 0.82, *p* = 0.001). On stepwise multivariate regression analysis, MRE was the only variable independently associated with HVPG (*R*^2^ = 0.377, *p* = 0.02).* Conclusions.* MRE of the liver correlated independently with HVPG. MRE is a valid noninvasive measure of liver disease severity and may prove to be a useful tool for noninvasive portal hypertension assessment.* Trial Registration Number*. This trial is registered with NCT01707472.

## 1. Background

Chronic liver injury with progressive hepatic fibrosis can result in cirrhosis, a leading cause of morbidity and mortality worldwide [1]. Portal hypertension, defined as increased pressure within the portal venous system, contributes to the serious complications of end stage liver disease, including gastroesophageal varices and ascites. The established standard for diagnosing portal hypertension is transjugular measurement of the hepatic venous portal gradient (HVPG), the difference between wedged and free hepatic venous pressures. HVPG measurements are utilized in managing liver disease for prognostication, monitoring of progression, and measuring response to therapy [2–5]. However, HVPG measurements are invasive, costly, and often available only at referral centers [6].

Identifying reliable noninvasive markers of portal hypertension would improve clinical ability to determine disease status and monitor fibrosis progression with reduced risk compared with HVPG measurements. A number of laboratory tests and laboratory indices have been evaluated as noninvasive markers of portal hypertension, including platelet count, prothrombin time, and aspartate aminotransferase platelet ratio (APRI); however these markers cannot reliably predict the presence of varices or serve as surrogates for HVPG [7].

Conventional imaging modalities have been used to support a diagnosis of cirrhosis and portal hypertension but have limited utility for confirming the presence of fibrosis or for the longitudinal assessment of portal hypertension [8–10]. Ultrasound-based vibration-controlled transient elastography (VCTE; Fibroscan) can predict advanced fibrosis and cirrhosis and correlates with HVPG [11]. Liver stiffness measured by VCTE predicts clinical decompensation and complications of portal hypertension [12]. However, VCTE is limited by operator dependence, small sampling window, and unreliability in patients with abdominal obesity or ascites [13].

Magnetic resonance elastography (MRE) is now available as an alternative method for determining liver stiffness. MRE can accurately detect both early and advanced fibrosis [14] and predict the presence of esophageal varices [15]. MRE can be successfully performed in most patients, including those with ascites, anatomical variants, and liver transplant recipients [16]. The accuracy of MRE appears to be superior to VCTE [17, 18], though the number of clinical studies that compare the two methods is small. The use of MRE of the liver in assessment of portal hypertension has been explored in animal models [19]; however studies in humans are limited to the detection of clinical manifestations of portal hypertension, such as the development of esophageal varices [20].

In this prospective, exploratory study, we assessed the accuracy of noninvasive fibrosis biomarkers and MRE shear wave velocity for the diagnosis of fibrosis and portal hypertension in a cohort of hepatitis C (HCV) and/or human immunodeficiency virus- (HIV-) infected liver disease patients. Participants were evaluated as part of a clinical trial evaluating the safety and efficacy of simtuzumab (Gilead Sciences, Inc., Foster City, CA), a humanized monoclonal antibody that inhibits lysyl oxidase-like 2 (LOXL2), an enzyme that contributes to liver fibrosis by catalyzing collagen cross-linkage.

## 2. Methods

### 2.1. Patient Population

Adults 18 years of age or older, with HIV, HCV, or HIV/HCV, were enrolled from October 2012 to December 2014. HCV infection was confirmed by measurement of HCV RNA >2,000 IU/mL. For HIV-infected subjects, an HIV viral load <400 copies/mL on stable combination antiretroviral therapy for ≥3 months was required. Patients with known cirrhosis could participate if they were Child-Pugh class A [21].

The NIAID Institutional Review Board approved the study. All participants provided written informed consent (NCT01707472). The primary study results are reported elsewhere [22].

### 2.2. Transjugular Liver Biopsy with Portal Pressure Measurements

HVPG measurements were obtained during transjugular liver biopsy. The left, middle, and right hepatic veins were cannulated and the free hepatic vein pressure (FHVP) and the wedged hepatic venous pressure (WHVP) were assessed. The WHVP was measured within each hepatic vein branch with gentle inflation of a balloon occlusion catheter. HVPG was calculated by subtracting the FHVP from the WHVP. Peak HVPG is reported and used for analysis.

### 2.3. Histology

Formalin-fixed paraffin-embedded liver biopsy sections were stained with hematoxylin and eosin, Masson's trichrome, or reticulin and interpreted by a single liver pathologist (DEK). Inflammatory activity and fibrosis were scored using the modified histology activity index (Ishak) scoring system [23]. Steatosis was graded on a scale of 0 to 3 based on the percentage of cells with fat according to the NASH-Clinical Research Network scoring system [24]. Stellate cell activation was quantified by immunohistochemical staining for activated smooth muscle actin [25] (Inova Health Systems, Falls Church, VA).

### 2.4. Magnetic Resonance Elastography

The imaging protocol for magnetic resonance elastography (MRE) has been described previously [26]. Briefly, MR examination was performed on a 3.0T system (Achieva, Philips Medical Systems, Best, Netherlands) using 32-element surface coil. A mechanical transducer set at vibration frequency of 56 Hz was placed against the supine subjects' side at the lowest right rib in the right-left direction. An operator blinded to participant clinical status performed image processing to provide shear wave speed in m/sec.

### 2.5. Laboratory Markers of Fibrosis

Platelet counts, liver-associated enzymes including alkaline phosphatase, aspartate aminotransferase (AST), alanine aminotransferase (ALT), and gamma-glutamyl transferase (GGT), and noninvasive estimators of liver fibrosis validated in similar liver disease populations, including the APRI [27], FIB-4 [28], Forns [29], and Fibroindex [30], were determined at the time of liver biopsy.

### 2.6. Soluble LOXL2

Soluble LOXL2 (sLOXL2) concentrations were measured in serum using a proprietary immunoassay (Singulex, Alameda, CA).

### 2.7. Hepatic LOXL2 Gene Expression

In a subset of participants (*n* = 12), total RNA isolated from liver was reverse transcribed using random primers with the High Capacity cDNA Reverse Transcriptase Kit (ThermoFischer Scientific, Waltham, MA), as previously described [22, 31]. Gene expression was determined as cycle of threshold (Ct) based on 40 PCR cycles, using expression of* GAPDH* and* GUSB* as endogenous controls to determine delta Ct values.* GAPDH* Ct values were distributed between 23 and 27. Data from 2 samples was excluded from analysis due to inadequate signal strength, defined as a* GAPDH* Ct value >27. Thus, confirmatory qRT-PCR data are presented from 10 of 12 subjects. Expression reactions using predesigned Taqman assays assembled into custom-designed 96-well plates (ThermoFischer Scientific) were run on an Applied Biosystems 7500 Real-Time PCR System, as previously described [31].

### 2.8. Statistical Analysis

Pairwise correlations between biomarkers of interest were evaluated with Spearman's correlation coefficient. For this exploratory analysis, a *p* value of ≤0.05, without adjustment for multiple comparisons, was considered statistically significant. Simple linear regression was employed to screen for biomarkers associated with HVPG. Biomarkers with a *p* value ≤0.15 from the simple linear regressions were identified as potential candidates. Backward stepwise multiple regression analysis was performed on HVPG using the candidate biomarkers. Stepwise variable elimination was based on a threshold *p* value of ≤0.15. Analyses were performed using JMP v.11 (SAS, Cary, NC, USA).

### 2.9. Data Availability

Datasets analyzed for the current study are available from the corresponding author on request.

## 3. Results

### 3.1. Baseline Demographic and Clinical Characteristics

Twenty-three patients completed the screening evaluation. Demographic and clinical characteristics of the cohort are shown in Table 1. The median age was 57 years (range 45–76 years) and 78% of participants were males. HCV was present in 18 (78%), 9 of whom had HIV coinfection. Sixteen (89%) of the HCV-infected participants were genotype 1. Five (22%) participants had HIV infection and nonalcoholic steatohepatitis (NASH) [24].

Liver biopsy size ranged from 6 to 24 mm, median 12 mm. Six (26%) of samples were <10 mm and therefore considered suboptimal for staging and grading [32].

Median Ishak fibrosis score was 4 (range 1–6) and 11 participants (48%) had cirrhosis, all Child-Pugh class A. Median HVPG was 8 mmHg (range 3–16 mmHg) and HVPG was ≥10 mmHg in 8 (35%) participants.

### 3.2. Correlates of HVPG

HVPG (*n* = 23) correlated positively with AST (*r* = 0.48, *p* = 0.01) and GGT (*r* = 0.62, *p* = 0.001) and negatively correlated with platelets (*r* = −0.72, *p* = 0.002). No significant correlation was seen between HVPG and ALT (Table 2).

While HVPG correlated with liver biopsy fibrosis score (*r* = 0.52, *p* = 0.04), HVPG demonstrated a better correlation with Forns' Index (*r* = 0.76, *p* < 0.001), Fibroindex (*r* = 0.75, *p* = 0.001), and APRI (*r* = 0.59, *p* = 0.02). (Table 2).

Stepwise regression analysis, including AST, GGT, platelets, liver biopsy fibrosis score, and MRE, identified MRE as the only biomarker independently associated with HVPG (*R*^2^ = 0.377, *p* = 0.015).

### 3.3. Correlates of MRE

**Figure 1:**
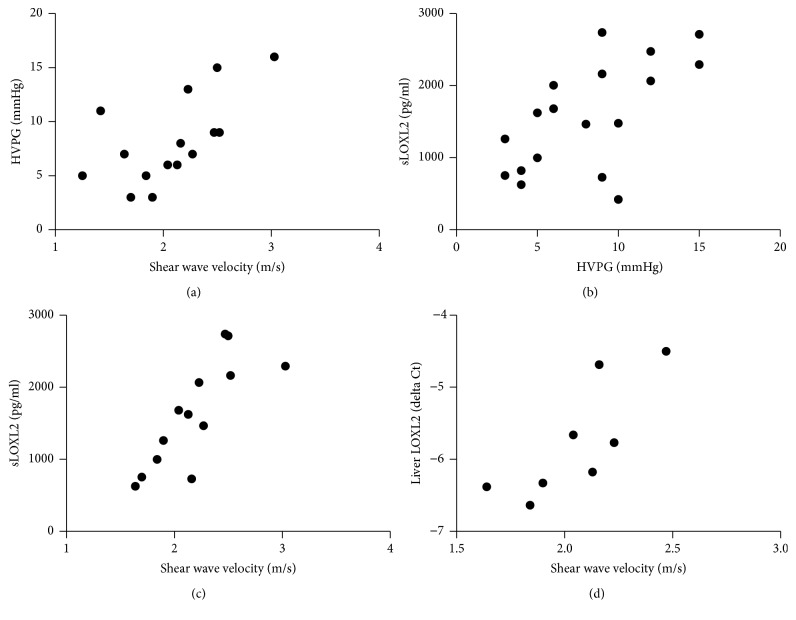
Significant correlations were seen between MRE-measured shear wave velocity, a measure of liver stiffness, and HVPG (a), sLOXL2 (c), and liver LOXL2 (d). sLOXL2 also correlated well with HVPG (b).

MRE was completed in 15 participants (3 HCV, 7 HIV/HCV, and 5 HIV/NASH). Median shear wave velocity was 2.13 m/sec (range 1.25–3.03 m/sec). MRE correlated significantly with HVPG (*r* = 0.64, *p* = 0.009; Figure 1), as well as with Ishak fibrosis score (*r* = 0.71, *p* = 0.003), total histologic activity index (HAI) inflammation (*r* = 0.64, *p* = 0.01), periportal inflammation (*r* = 0.72, *p* = 0.002), lobular inflammation (*r* = 0.8, *p* = 0.002), and *α*SMA (*r* = 0.70, *p* = 0.008) (Table 2).

Furthermore, MRE had a significant positive correlation with AST (*r* = 0.74, *p* = 0.002) and significant negative correlation with platelets (*r* = −0.70, *p* = 0.004). In addition, MRE had significant correlations with noninvasive fibrosis biomarkers, including APRI (*r* = 0.81, *p* < 0.001), FIB-4 (*r* = 0.67, *p* = 0.006), Fibroindex (*r* = 0.76, *p* = 0.001), and Forns' Index (*r* = 0.72, *p* = 0.002). No correlation with ALT (*r* = 0.28, *p* = 0.31) or GGT (*r* = 0.43, *p* = 0.1) was observed.

### 3.4. Correlates of LOXL2

Soluble LOXL2 (*n* = 23) levels correlated significantly with AST (*r* = 0.70, *p* = 0.001) and negatively with platelets (*r* = −0.57, *p* = 0.01) (Table 2). sLOXL2 also had significant positive correlation with the noninvasive fibrosis biomarkers, APRI (*r* = 0.72, *p* = 0.001), FIB-4 (*r* = 0.71, *p* = 0.001), Fibroindex (*r* = 0.80, *p* = <0.0001), and Forns' Index (*r* = 0.6, *p* = 0.009).

Soluble LOXL2 correlated with HVPG (*r* = 0.58, *p* = 0.02) but did not correlate with fibrosis or inflammation by biopsy.

Liver LOXL2 gene expression, analyzed by PCR in 10 participants, also correlated with HVPG (*r* = 0.69, *p* = 0.03) and HAI total (*r* = 0.82, *p* = 0.006), but not with fibrosis (*r* = 0.18, *p* = 0.61).

Serum soluble (*n* = 13; *r* = 0.82, *p* < 0.001) and liver LOXL2 (*n* = 8; *r* = 0.82, *p* = 0.03) both had strong correlations with MRE (Figure 1).

## 4. Discussion

We have demonstrated that hepatic MRE measurement of shear wave velocity may be a valuable biomarker in assessing the degree of portal hypertension as measured by HVPG. Stepwise regression analysis of HVPG on multiple biomarkers of interest showed that MRE had an independent association with HVPG, suggesting potential utility of MRE in detection and monitoring of portal hypertension. Additionally, our study demonstrates correlations between MRE-measured hepatic shear wave velocity with other noninvasive fibrosis biomarkers, including APRI, FIB-4, Fibroindex, and Forns' Index, and with sLOXL2 levels, although none of these markers showed an independent association with HVPG in a multivariate analysis.

In porcine and canine models of progressive portal hypertension, liver and spleen stiffness assessed by MRE correlated with portal pressure [19, 33]. In a small human study, MRE was sensitive to pressure when change in volumetric strain was found after transjugular intrahepatic portosystemic shunt (TIPS) placement [34]. This study demonstrated that percentage change in volumetric strain measurements before and after TIPS procedure correlated well with pre-TIPS HVPG. These findings suggest that MRE measures dynamic, pressure-dependent liver stiffness in addition to the static components of fibrosis. However, to the best of our knowledge, our study is the first to identify a strong correlation between MRE-measured shear wave velocity, HVPG, and serum soluble LOXL2. This was possible at higher magnetic field strength (3 T) not used in the previously mentioned studies. A higher magnetic field is known to be more sensitive to phase changes that are the backbone of the MRE readout sequences. Additionally, utilizing 32-channel coils and mechanical vibrations (instead of acoustic vibrations) might have added to the robustness and sensitivity of our methods.

MRE shear wave velocity also correlated with hepatic fibrosis, confirming the findings of earlier MRE studies in HCV and nonalcoholic fatty liver disease [35–37]. Interestingly, MRE had a strong correlation with histologic inflammatory scores and *α*SMA in addition to histologic fibrosis, suggesting that MRE-measured shear wave velocity may detect hepatic inflammation. Studies have shown hepatic inflammation can increase liver stiffness irrespective of fibrosis [38], although the mechanism is not fully understood. However, the increase in cellular volume seen with inflammation may affect the procession of the shear wave as it propagates through the hepatic tissue. The dynamic component of liver stiffness is affected by change in perfusion that can be influenced by hepatic inflammation. Moreover, changes in the mechanical state of cells capable of contraction, such as vascular smooth muscle cells and activated hepatic stellate cells in the perisinusoidal spaces, can influence liver stiffness [39].

A correlation was also seen between *α*SMA, a contractile protein expressed by activated hepatic stellate cells with the myofibroblast phenotype, and HVPG. *α*SMA is increased in the liver of patients with chronic liver diseases and correlates with the extent of hepatic fibrosis [40]. In chronic viral hepatitis, *α*SMA also correlates with necroinflammatory grade, suggesting that hepatocyte necroinflammation drives hepatic stellate cell activation and subsequent fibrogenesis [41]. Our observations agree with accumulating evidence suggesting that stellate cells also regulate liver microcirculation and portal pressure. In animal models of fibrosis, *α*SMA causes cellular contractility through calcium dependent and independent contractile forces, leading to increased portal resistance [42]. As fibrosis advances, myofibroblasts are recruited and impede portal circulation through vasoactive mediators and interactions with the extracellular matrix [43, 44]. Inhibition of *α*SMA with nitric oxide in rat and human hepatic stellate cells reduces portal pressure by 20% [45].

We also observed a correlation between sLOXL2 levels, hepatic LOXL2 expression, and portal pressure. In addition to cross-linking collagen, LOXL2 increases matrix tension, triggering fibroblasts to covert to contractile myofibroblasts through activation of hepatic TGF*β*1 [46, 47]. LOXL2 also stimulates expression of myofibroblasts grown on collagen matrices through integrin mediated focal adhesion kinase activation [48]. As myofibroblasts regulate portal resistance, LOXL2 may also contribute to development of portal hypertension. Interestingly, sLOXL2 had the most significant correlation with MRE compared to other parameters. Longitudinal studies of hepatic fibrosis, including ongoing trials of simtuzumab in NASH and advanced liver fibrosis, will contribute to the understanding of the function of LOXL2 in fibrosis progression and portal hypertension.

Limitations of our study include the cross-sectional design, small size with limited numbers of patients with cirrhosis and portal hypertension, and lack of a validation cohort. Further cross-sectional and longitudinal study in a larger population of patients with clinically significant portal hypertension is needed to confirm our findings. Multiple comparisons were made and, though statistically significant, many correlations were weak. Additionally, the liver biopsy samples obtained by transjugular biopsy were small, with 6 (26%) suboptimal for grading, likely resulting in understaging and undergrading of fibrosis [32] and potentially attenuating relationships between biopsy parameters and noninvasive markers. MRE requires specialized hardware, usually only available at academic medical centers, and is limited to patients eligible and tolerant of MR imaging. Compared to transient elastography and other ultrasound-based shear wave elastography methods, MRE would be expected to cost more.

In conclusion, MRE shear wave velocity correlates with noninvasive biomarkers and was independently associated with HVPG. Given this, MRE may prove to be a useful noninvasive measure of disease severity and portal hypertension. In addition, our study demonstrated the novel correlation of soluble LOXL2 and hepatic LOXL2 expression with portal hypertension.

## Figures and Tables

**Table 1:** Baseline demographic and clinical characteristics of study subjects (*n* = 23).

Parameter	
Age, years	54 (45–76)
Male, *n* (%)	18 (78%)
Liver disease etiology, *n* (%)	
HCV	9 (39%)
HCV/HIV	9 (39%)
HIV/NASH	5 (22%)
Body mass index, kg/m^2^	30 (21–46)
>30 kg/m^2^ (obesity), *n* (%)	12 (52%)
Laboratory studies	
Platelets, K/uL	159 (45–284)
Alkaline phosphatase, U/L	107 (51–210)
Aspartate aminotransferase (AST), U/L	56 (22–151)
Alanine aminotransferase (ALT), U/L	77 (30–161)
Total bilirubin, mg/dL	0.8 (0.3–2.3)
Direct bilirubin, mg/dL	0.3 (0.1–1.4)
Gamma-glutamyl transferase (GGT), U/L	150 (19–531)
Albumin, g/dL	4.1 (3.0–5.5)
Prothrombin time (PT), seconds	14.3 (12.3–16.4)
International normalized ratio (INR)	1.1 (0.9–1.3)
Hepatitis C characteristics (*n* = 18)	
HCV viral load, log⁡10, IU/mL	6.9 (4.7–7.8)
Hepatitis C genotype, *n* (%)	
1a	13 (72)
1b	3 (17)
2	1 (6)
4	1 (6)
MRE shear wave velocity, m/sec (*n* = 15)	2.13 (1.25–3.03)
HVPG, mmHg	6 (3–16)
Liver biopsy length, mm	12 (6–24)
<10 mm, *n* (%)	6 (26)
Liver biopsy scoring	
Fibrosis, Ishak (range 0–6)	4 (1–6)
Inflammation, total HAI (range 0–18)	8 (1–14)
Steatosis (range 0–4)	1 (0–2)

Median, range presented unless otherwise noted.

**Table 2:** Correlation coefficients (Spearman *ρ*) for selected variables^*∗*^.

	MRE shear wave velocity	HVPG	Ishak fibrosis score	sLOXL2
MRE shear wave velocity (*n* = 15)		*0.64*	0.71	0.82
HVPG (*n* = 23)	*0.64*		0.53	*0.58*
Liver biopsy (*n* = 23)				
Ishak fibrosis score	0.71	0.53		0.31
Total HAI inflammation score	*0.64*	*0.49*	0.36	0.30
% alpha smooth muscle actin (*α*SMA)	0.74	*0.50*	*0.51*	0.08
Selected laboratory studies (*n* = 23)				
Platelets	−0.70	−0.72	*−0.47*	*−0.57*
Aspartate aminotransferase (AST)	0.74	*0.50*	0.55	0.70
Alanine aminotransferase (ALT)	0.28	0.13	*0.42*	0.24
Gamma-glutamyl transferase (GGT)	0.43	0.63	0.56	0.28
Non-invasive fibrosis indices (*n* = 23)				
APRI	**0.81**	0.57	0.57	**0.72**
FIB-4	0.67	**0.66**	*0.44*	0.71
Forns' index	0.71	**0.76**	0.44	*0.60*
Fibroindex	0.75	**0.75**	0.40	**0.80**
Serum soluble LOXL2 (*n* = 23)	0.82	*0.58*	0.31	
Liver LOXL2 (*n* = 10)	*0.86* ^a^	*0.69*	0.18	0.31

MRE: magnetic resonance elastography; HVPG: hepatic venous pressure gradient; HAI: histologic activity index; APRI: AST/platelet ratio index; LOXL2: lysyl oxidase-like 2.

^a^MRE and liver LOXL2 results available for 8 participants.

^*∗*^ *p* values are indicated as follows: *Italics*, 0.01–0.05; Underlined, 0.001–<0.01; **Bold**, <0.001.

## Abbreviations

*α*SMA: Alpha smooth muscle actin
ALT: Alanine aminotransferase
AST: Aspartate aminotransferase
APRI: Aspartate aminotransferase to platelet ratio index
FHVP: Free hepatic vein pressure
GGT: Gamma-glutamyl transferase
HAI: Histologic activity index
HCV: Hepatitis C virus
HIV: Human immunodeficiency virus
HVPG: Hepatic venous pressure gradient
LOXL2: Lysyl oxidase-like 2
MRE: Magnetic resonance elastography
NASH: Nonalcoholic steatohepatitis
qRT: Quantitative real time
RNA: Ribonucleic acid
TGF*β*1: Transforming growth factor beta-1
TIPS: Transjugular intrahepatic portosystemic shunt
WHVP: Wedged hepatic venous pressure
VCTE: Vibration-controlled transient elastography.
